# A Novel Mutation in the Fibrinogen Bβ Chain (c.490G>A; End of Exon 3) Causes a Splicing Abnormality and Ultimately Leads to Congenital Hypofibrinogenemia

**DOI:** 10.3390/ijms18112470

**Published:** 2017-11-20

**Authors:** Chiaki Taira, Kazuyuki Matsuda, Shinpei Arai, Mitsutoshi Sugano, Takeshi Uehara, Nobuo Okumura

**Affiliations:** 1 Department of Health and Medical Sciences, Graduate School of Medicine, Shinshu University, 3-1-1 Asahi, Matsumoto 390-8621, Japan; nobuoku@shinshu-u.ac.jp; 2 Department of Laboratory Medicine, Shinshu University Hospital, Matsumoto 390-8621, Japan; kmatsuda@shinshu-u.ac.jp (K.M.); m061201h@shinshu-u.ac.jp (S.A.); suga@shinshu-u.ac.jp (M.S.); 3 Department of Laboratory Medicine, Graduate School of Medicine, Shinshu University, 3-1-1 Asahi, Matsumoto 390-8621, Japan; tuehara@shinshu-u.ac.jp

**Keywords:** hypofibrinogenemia, *FGB*, splicing abnormality, cryptic splice site

## Abstract

We found a novel heterozygous mutation in the fibrinogen Bβ chain (c.490G>A) of a 3-year-old girl with congenital hypofibrinogenemia. To clarify the complex genetic mechanism, we made a mini-gene including a *FGB* c.490G>A mutation region, transfected it into a Chinese Hamster Ovary (CHO) cell line, and analyzed reverse transcription (RT) products. The assembly process and secretion were examined using recombinant mutant fibrinogen. Direct sequencing demonstrated that the mutant RT product was 99 bp longer than the wild-type product, and an extra 99 bases were derived from intron 3. In recombinant expression, a mutant Bβ-chain was weakly detected in the transfected CHO cell line, and aberrant fibrinogen was secreted into culture media; however, an aberrant Bβ-chain was not detected in plasma. Since the aberrant Bβ-chain was catabolized faster in cells, the aberrant Bβ-chain in a small amount of secreted fibrinogen may catabolize in the bloodstream. *FGB* c.490G>A indicated the activation of a cryptic splice site causing the insertion of 99 bp in intron 3. This splicing abnormality led to the production of a Bβ-chain possessing 33 aberrant amino acids, including two Cys residues in the coiled-coil domain. Therefore, a splicing abnormality may cause impaired fibrinogen assembly and secretion.

## 1. Introduction

Fibrinogen is a 340-kDa plasma glycoprotein consisting of a hexameric molecule of three polypeptide chains: Aα-, Bβ-, and γ-chains, which are synthesized and assembled into a six-chain molecule in hepatocytes, and secreted into the blood of healthy adults at a plasma concentration of 1.8–3.5 g/L [1,2]. Aα-, Bβ-, and γ-chains are composed of 610, 461, and 411 residues, which are encoded by *FGA* (five exons), *FGB* (eight exons), and *FGG* (10 exons), respectively, and are clustered in a 50-kb region on chromosome 4q31 [3].

Congenital fibrinogen disorders have been classified into four different manifestations: afibrinogenemia, hypofibrinogenemia, dysfibrinogenemia, and hypodysfibrinogenemia. Afibrinogenemia (the absence of fibrinogen) or hypofibrinogenemia (low plasma levels of fibrinogen) has been defined as a reduction in the quantity of fibrinogen in plasma. Dysfibrinogenemia has been defined as a qualitative abnormality a low functional activity and a normal antigenic fibrinogen. Hypodysfibrinogenemia is rarer and has the characteristics of both dysfibrinogenemia and hypofibrinogenemia [4,5]. Patients with hypofibrinogenemia or dysfibrinogenemia have a higher risk of bleeding during surgery or pregnancy than the general population [6].

In the past three decades, more than 350 genetic abnormalities in patients with congenital fibrinogen disorders have been detected in three genes: *FGA*, *FGB*, and *FGG*, as listed in the fibrinogen variant database [7]. Homozygosity or heterozygosity mutations including missense mutations, nonsense mutations, frameshift mutations, splice-site abnormalities, and large deletions have been identified in patients with afibrinogenemia or hypofibrinogenemia. On the other hand, most patients with dysfibrinogenemia have heterozygous missense mutations leading to delayed or absent fibrinopeptide A release or defective fibrin polymerization causing a bleeding disorder or thrombophilia [4]. It is important to characterize these mutations for the diagnosis, confirmation, or identification of potential carriers and a familial diagnosis.

We recently found a new heterozygous mutation in the fibrinogen Bβ chain (c.490G>A) of a 3-year-old girl with congenital hypofibrinogenemia. In order to clarify the complex genetic mechanism for this novel *FGB* mutation (designated as Kyoto IX), we made a mini-gene including a point mutation region, transfected it into a Chinese Hamster Ovary (CHO) cell line, and analyzed reverse transcriptase-polymerase chain reaction (RT-PCR) products. In addition, we established a recombinant fibrinogen-producing CHO cell line with the *FGB* c.490G>A mutation. Using this recombinant mutant fibrinogen, the assembly process and secretion of mutant fibrinogen were analyzed.

## 2. Results

### 2.1. Patient with Kyoto IX

The patient was a three-year-old girl with adenoidal hypertrophy. She and her family members showed no bleeding or thrombotic episodes, and her coagulation test results were as follows: PT 13.4 s, PT-INR 1.17 (normal range: 0.85–1.15), APTT 30.5 s (normal range: 23.0–38.0 s), and functional and antigenic fibrinogen concentrations: 52.0 and 68.4 mg/dL (normal range: 180–350 mg/dL).

### 2.2. DNA Sequence Analysis of the Kyoto IX Patient

The nucleotide sequence of the Aα-, Bβ-, γ-chain gene-coding regions, including exon–intron boundaries, was elucidated by a direct sequence analysis. The sequence showed a heterozygous G > A single nucleotide mutation at position 490 (c.490G>A) in *FGB*. We also analyzed the nucleotide sequence of exon 3 in the *FGB* of Kyoto IX parents, and the father had the same mutation (c.490G>A) as the patient. On the other hand, the mother had the wild-type nucleotide.

### 2.3. Characterization of Plasma Fibrinogen

**Figure 1:**
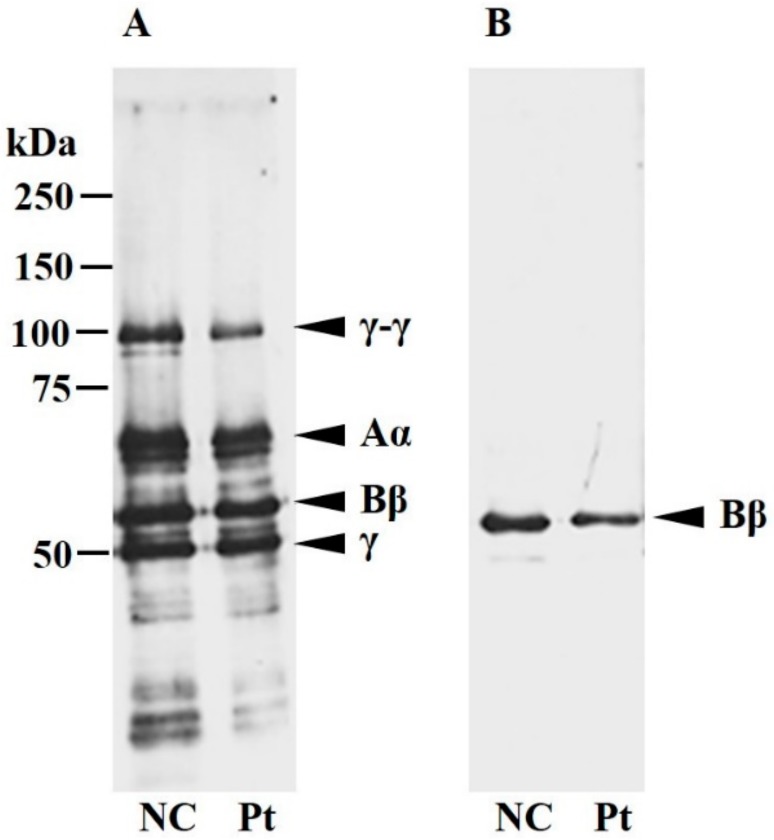
Western blot analysis of plasma fibrinogen. A healthy volunteer’s plasma (NC) and the patient’s plasma (Pt), equivalent to 100 ng fibrinogen, were separated on 10% SDS-PAGE under reducing conditions. Blots were developed with an anti-fibrinogen polyclonal antibody (**A**) or anti-Bβ-chain polyclonal antibody (**B**) as described in the Materials and Methods section. The bands derived from normal fibrinogen are indicated as γ-γ-, Aα-, Bβ-, and γ-chains, and the patient band pattern corresponded to NC.

We performed a Western blot analysis on Kyoto IX plasma fibrinogen (Figure 1). Under reducing conditions, four bands compatible with the normal γ-γ-, Aα-, Bβ-, and γ-chains were detected in Kyoto IX fibrinogen when an anti-human fibrinogen antibody was used. When an anti-human fibrinogen Bβ-chain antibody was used, the single band position in Kyoto IX fibrinogen was concordant with the normal Bβ-chain band in the plasma fibrinogen of a healthy volunteer (normal control, NC).

### 2.4. Analysis of FGB Gene Transcripts in the CHO Cell Line

**Figure 2:**
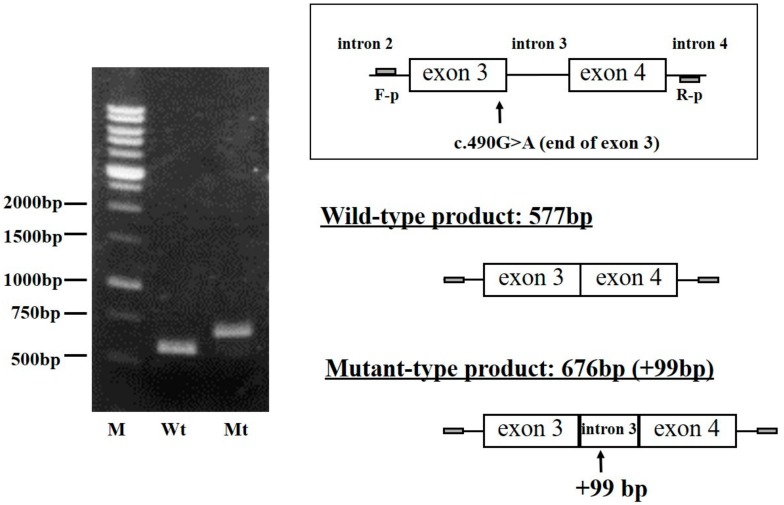
Analysis of fibrinogen Bβ-chain mini-gene transcripts in CHO cell lines. The PCR-amplified mini-genes of the Kyoto IX propositus were cloned into the pSecTaq/FRT/V5-His TOPO^®^ vector and transfected as described in the Materials and Methods section. ***Upper right***: Construction of the mini-gene by amplification with the forward primer (F-p) and reverse primer (R-p). ***Left***: RT-PCR products were separated on a 1% agarose gel. ***Lower right***: Schematic structure predicted from sequencing for RT-PCR-amplified mini-gene products. Mini-gene was constructed from intron 2 to intron 4. Wt: RT-PCR amplified products from wild-type mini-gene-derived mRNA. Mt: RT-PCR amplified products from aberrant mini-gene-derived mRNA. M: Molecular size markers.

In order to verify whether the *FGB* c.490G>A mutation influences the transcription of mature mRNA, mutant Bβ-chain mRNA was transiently produced in the CHO cell line and analyzed as described in the Materials and Methods section. After RNA extraction and RT-PCR, the mini-gene incorporating the *FGB* c.490G>A mutation (mutant-type, Mt) and the wild-type (Wt) mini-gene were amplified by the primer sets designed in introns 2 and 4. The results of electrophoresis showed that the Mt band was larger than the Wt band (Figure 2). Direct sequencing demonstrated that the Mt product was 676 bp and, thus, 99 bp longer than the Wt product (577 bp), and the extra 99 bases were derived from intron 3.

**Figure 3:**
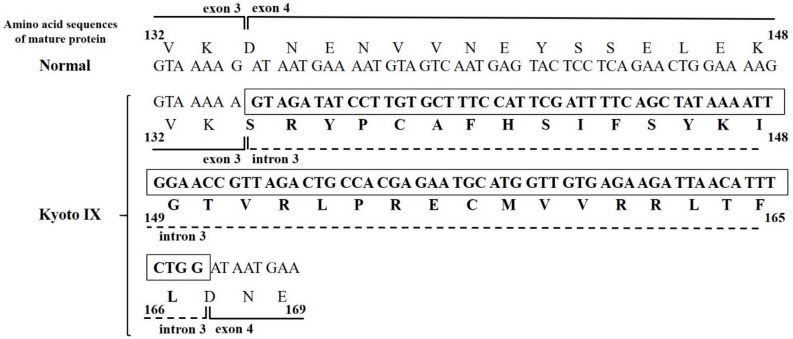
Predicted amino acid sequences for the mature protein of the Kyoto IX propositus. The aberrant amino acid sequences of Bβ-combined with exon 3, intron 3, and exon 4 are indicated. Bold letters in the box indicate aberrant nucleotide sequences and bold letters without the box indicate aberrant amino acid sequences.

We showed the predicted amino acid sequences of Kyoto IX from mini-gene expression results (Figure 3). The splicing abnormality caused by the *FGB* c.490G>A mutation led to the production of a Bβ-chain possessing 33 aberrant amino acids between Lys133 and Asp134 (mature protein).

### 2.5. Synthesis and Secretion of Recombinant Variant Fibrinogen in CHO Cell Lines

**Figure 4:**
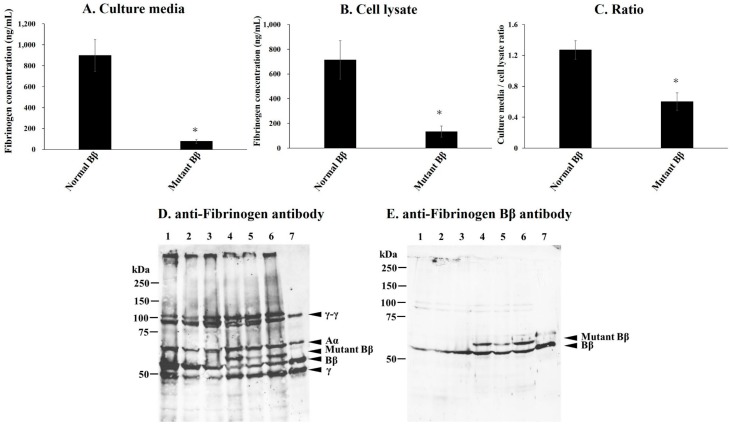
Synthesis of variant fibrinogen in transfected CHO cell lines. Fibrinogen concentrations in culture media (**A**) and cell lysates (**B**) were measured by ELISA as described in the Materials and Methods section. The ratios of the values of the culture media to the cell lysate are shown in (**C**). Mean values are presented with standard deviations indicated by error bars for Normal- Bβ-Fbg: *n* = 3, Aberrant- Bβ-Fbg: *n* = 3. The culture media, cell lysate, and ratio of the aberrant were significantly different from the normal (*: *p* < 0.05). (**D**,**E**): A Western blot analysis of cell lysates. Samples from cell lysates were subjected to 10% SDS-PAGE under reducing conditions. Blots were reacted with an anti-fibrinogen antibody (**D**) and anti-fibrinogen Bβ antibody (**E**), and detected by chemiluminescence as described in the Materials and Methods section. Lanes 1–3: Normal Bβ-Fbg synthesized in CHO cell lines, Lanes 4–6: Aberrant Bβ-Fbg synthesized in CHO cell lines, Lane 7: purified fibrinogen from normal plasma. The left sides of Panels D and E show molecular size markers.

We established a mutant-Bβ-fibrinogen-expressing CHO cell line (*n* = 3) and normal-Bβ-fibrinogen-expressing CHO cell line (*n* = 3). Fibrinogen concentrations in the culture media and cell lysates of the fibrinogen-synthesizing cell lines were measured using an enzyme-linked immunosorbent assay (ELISA). Fibrinogen concentrations as the mean ± SD and range in the culture media from the mutant-Bβ- and normal-Bβ-fibrinogen-expressing CHO cell lines were 79 ± 20 (56–90) ng/mL and 899 ± 154 (800–1076) ng/mL, respectively (Figure 4A). Fibrinogen concentrations in cell lysates from the mutant-Bβ- and normal-Bβ-fibrinogen-expressing CHO cell lines were 134 ± 46 (93–183) ng/mL and 714 ± 155 (582–885) ng/mL, respectively (Figure 4B). The fibrinogen concentration ratios of the culture media/cell lysates of the mutant-Bβ- and normal-Bβ-fibrinogen-expressing CHO cell lines were 0.60 ± 0.11 (0.49–0.71) and 1.27 ± 0.12 (1.22–1.41), respectively (Figure 4C).

Cell lysates were analyzed by sodium dodecyl sulfate-polyacrylamide gel electrophoresis (SDS-PAGE) and a Western blot analysis under reducing conditions, as described in the Materials and Methods section. Aberrant bands, the relative molecular weights of which were higher than the normal Bβ-chain, were shown in the mutant-Bβ-fibrinogen-expressing CHO cell line (Figure 4D). These aberrant bands reacted with the anti Bβ-chain antibody (Figure 4E), and possibly with the mutant Bβ-chain possessing 33 aberrant amino acids. However, bands with similar molecular weights to the normal Bβ-chain were also detected in the mutant-Bβ-fibrinogen-expressing CHO cell line (Figure 4D,E), and these may be degradation products derived from the mutant Bβ-chain.

## 3. Discussion

We identified a novel heterozygous point mutation, c.490G>A, in *FGB* in a 3-year-old girl with congenital hypofibrinogenemia (Kyoto IX). Plasma fibrinogen from Kyoto IX had a normal pattern of Aα-, Bβ-, and γ-chains in a Western blot analysis; there was no aberrant Bβ-chain in her plasma. Therefore, we analyzed mRNA sequences derived from a mini-gene of Kyoto IX, and the assembly process and secretion of mutant fibrinogen using recombinant fibrinogen with c.490G>A in *FGB*.

*FGB* c.490G>A is a point mutation at the end of exon 3, right next to the 5′ splice donor site. We initially predicted the existence of a simple missense mutation (Asp134Asn) from the results of plasma fibrinogen Western blotting. We then predicted that the splicing error resulted in a translational frameshift and premature termination signal within intron 3, leading to transcript elimination and shorter products than wild-type transcriptional products (Asp134Ser fs33aa-stop) [8,9]. However, our mutant mini-gene with c.490G>A expressed transcripts that were longer than wild-type mini-gene-expressed transcripts by PCR amplification (Figure 2). The results of sequencing analysis showed that the mutation destroyed the splice donor site and yielded a newly spliced product maintaining the original reading frame. Furthermore, the activation of a cryptic splice site in intron 3 and the skip to the 3′ splice-acceptor site intron 3 resulted in the aberrant transcriptional products composed from exon 3, partial intron 3 (99 bp), and exon 4. This result was consistent with that of Splice Site Prediction by the Neural Network (http://www.fruitfly.org/seq_tools/splice.html). The *FGB* mutations reported to date are mostly missense and nonsense mutations on the insides of exons [7]. On the other hand, few splice-site and frameshift mutations have been identified; all 7 splice-site mutations were in introns only, while all 3 frameshift mutations were deletions and/or insertions [10]. c.490G>A was novel and rare because of the end basal mutation in exon 3 leading to a splice-site abnormality. This is the first study on fibrinogen genes where a mutation at the end of an exon leads to the activation of a cryptic splice site.

In *FGB*, most of the reported missense and nonsense mutations are localized in the highly conserved globular C-terminal domains of the Bβ-chain, which were previously shown to specifically impair fibrinogen secretion [10]. Our aberrant transcript, Asp134Ser, was a predicted insertion of 33 amino acids derived from intron 3. This region is a coiled-coil domain, and the 33-residue addition may lengthen the Bβ coiled-coil. The deletion of five residues of the coiled-coil domain in the Bβ-chain was previously reported by Brennan SO et al. (Asn137_Glu141del) [11]. As well as the deletion, an addition may result in tension and deform the super coil, changing the distance between the E and D domains. This may be reflected in aberrant polymerization. A missense mutation (Met118Lys) of the initial portion of the coiled-coil domain in the Bβ-chain [12] also led to a conformational change in the fibrinogen molecule by replacing the non-polar residue with the polar residue that may alter the fibrinogen assembly. N-terminal disulfide bonds (AαCys28-AαCys28, γCys8-γCys8, γCys9-γCys9) are important in the docking of the two Aα-Bβ-γ half-molecules for maintain structural integrity. Furthermore, a pair of disulfide ring at either end of each coiled-coil domains of Aα-, Bβ-, and γ-chain are also important in stabilization of fibrinogen molecule [13,14]. Therefore, the disulfide bond mutation, AαCys45Tyr [15] or AαCys45Phe [16], prevents half-molecule dimerization and causes an impairment in intracellular fibrinogen processing. Our aberrant transcripts have two Cys residues as candidate disulfide bond sites. We speculate that the additional two Cys residues in the Bβ chain disturb the formation of disulfide rings or intrachain disulfide bonds in the coiled-coil domain, destroy the tertiary structure of the Bβ-chain and/or Aα-Bβ-γ half molecules, and ultimately impair the assembly and secretion of fibrinogen.

The mutant Bβ-chain was weakly detected in the transfected CHO cell lines, and was secreted to the culture media (Figure 4A–C). However, aberrant fibrinogen was not detected in plasma (Figure 1). In mutant Bβ recombinant expression, two bands were observed by an anti Bβ-chain antibody; one had a higher molecular weight than the normal Bβ-chain, while the other had a similar molecular weight. We considered the former band to be an aberrant Bβ-chain and the latter to be the cleavage product of the aberrant Bβ-chain (Figure 4C,D). The aberrant Bβ-chain was catabolized in cells, and a small amount of secreted fibrinogen containing the aberrant Bβ-chain may catabolize more rapidly in the bloodstream. Therefore, in plasma Western blotting, we speculate that there are a few aberrant Bβ-chains that are not detected, and the degradation product derived from the aberrant Bβ-chain migrated similarly to the normal Bβ-chain band.

In conclusion, the novel heterozygous hypofibrinogenemia, c.490G>A indicated the activation of a cryptic splice site causing the insertion of 99 bp in the intron 3 nucleotide. This mutation led to the insertion of 33 amino acids including two Cys residues, which may form a disulfide bond and ultimately disturb disulfide ring formation. These results suggest that a splicing abnormality that yields a newly spliced product maintaining the original reading frame leads to impaired fibrinogen assembly and secretion.

## 4. Materials and Methods

This study was approved by the Ethical Review Board of Shinshu University School of Medicine (#383, 4 September 2012). After informed consent had been obtained from the patient, blood samples were collected for biochemical and genetic analyses.

### 4.1. Characterization of Patient Plasma Fibrinogen

Patient plasma fibrinogen was analyzed by SDS-PAGE under reduced conditions (10% polyacrylamide gel) and immunoblots using a rabbit-anti-human fibrinogen antibody (DAKO, Carpinteria, CA, USA) or rabbit anti-human Bβ-chain antibody (Chemicon International, Temecula, CA, USA) [17]. Reacting species were visualized with a horseradish peroxidase-conjugated goat anti-rabbit IgG antibody (Medical and Biological Laboratories Ltd., Nagoya, Japan) and enhanced chemiluminescence (ECL) detection reagents (Amersham Pharmacia Biotech, Buckinghamshire, UK). Blots were then exposed on Hyperfilm-ECL (Amersham Pharmacia Biotech, Buckinghamshire, UK).

### 4.2. DNA Sequence Analysis

Genomic DNA was extracted from whole blood cells using a DNA Extraction Kit (WAKO Pure Chemical Ltd., Osaka, Japan), according to the manufacturer’s instructions. In order to amplify all exons and exon–intron boundaries in the Aα-, Bβ-, and γ-chain genes, 32 PCR primers were designed and DNA was amplified by PCR as described elsewhere [18]. PCR products were purified from agarose gels and directly sequenced using a BigDye^TM^ Terminator Cycle Sequencing Ready Reaction Kit (Thermo Fisher Scientific, Waltham, MA, USA) and 3500 Genetic Analyzer (Life Technologies, Carlsbad, CA, USA).

### 4.3. Construction of Mini-Gene and Expression Vectors

DNA fragments spanning from intron 2 to intron 4 of the *FGB* gene were amplified from patient and control genomic DNA using the primer couples *FGB*-IVS2-F (5′-CCTATGTGCTATTTTAACAAATGTCC-3′) and *FGB*-IVS4-R (5′-CACTTAGCATTTTTGTTGTTGTTG-3′). The mini-gene, a 1497-bp genomic fragment, was composed of part of intron 2 and intron 4, and all of exon 3, intron 3, and exon 4 in *FGB*. The purified PCR product of the mini-gene was inserted into the pSecTaq/FRT/V5-His TOPO^®^ vector and transfected into One Shot^®^ TOP10 Chemically Competent *E. coli* using the pSecTaq/FRT/V5-His TOPO^®^ TA Expression Kit (Invitrogen, Carlsbad, NM, USA), according to the manufacturer’s instructions. Plasmid DNAs were isolated using the QIAprep Spin Miniprep Kit (QIAGEN N.V., Hulsterweg, The Netherlands) and sequenced as described above. Two selected plasmids inserted by the wild- or mutant-type of *FGB* were cultured in a large amount of media, and plasmids were purified using a Qiagen Plasmid Maxi Kit (QIAGEN). The sequences of the plasmids were confirmed by direct sequencing according to the manufacturer’s instructions. The plasmids were named Bβ-wild-type (wt) and Bβ-mutant-type (mt) vectors.

### 4.4. Production of Bβ-Chain mRNAs, RNA Extraction, and RT-PCR

The expression vectors Bβ-wt and Bβ-mt were introduced into CHO cell lines using lipofection, as described previously [19,20]. Transfected CHO cell lines were cultured in 5% CO_2_ at 37 °C. CHO cell lines were harvested 48 h after transfection. Total cellular RNA was extracted from cells using the QIAamp RNA Blood Mini Kit (Qiagen), and contaminated DNA was digested using Recombinant DNase I (Takara Bio Inc., Kusatsu, Japan), according to the manufacturer’s instructions. Reverse-transcriptase (RT) reactions were performed in 20 µL of a reaction mixture containing 1 µg of extracted total RNA, 2 µL of RT buffer, 5 µL of 2.5 mmol/L dNTP mixture, 1 µL of 500 µg/mL oligo dT, 0.2 µL of 0.1 mol/L dithiothreitol, and 0.5 µL of 200 U/µL Moloney murine leukemia virus (M-MLV) RT at 42 °C for 1 h. After the RT reaction, cDNA was amplified by PCR using the two pairs of primers that were used in the PCR of the fragment DNA Bβ-chain gene under the following conditions: 30 cycles at 95 °C for 30 s, 60 °C for 1 min, and 72 °C for 2 min, preceded by 95 °C for 10 min, followed by 72 °C for 30 min. The amplified products were separated by electrophoresis on 1% agarose gels and purified from the gels using the Gene Clean II Kit (Funakoshi, Tokyo, Japan). DNA fragments were sequenced as described above using the primers that were used for the PCR of the patient’s genomic Bβ-chain gene.

### 4.5. Expression of Recombinant Mutant Fibrinogen

Recombinant mutant fibrinogens were prepared as described previously [21,22]. Briefly, the mutant Bβ-fibrinogen expression vector was prepared by the insertion of the 99-bp nucleotides of intron 3 into the pMLP-Bβ plasmid (kindly provided by Lord ST, University of North Carolina, Chapel Hill, NC, USA), which contained wild-type Bβ-chain cDNA, with restriction enzymes (Eco81I in exon 2 and Bst1107I in exon 5).

The resultant mutant and normal expression vectors were co-transfected with the histidinol selection plasmid (pMSVhis) into CHO cell lines that expressed normal human fibrinogen Aα- and γ-chains (Aαγ CHO cell-lines) using a standard calcium–phosphate co-precipitation method [23]. The cell lines were designated as mutant Bβ- and normal Bβ-CHO cell lines, respectively. Cells were cultured and colonies were selected on histidinol (Aldrich Chem. Co., Milwaukee, WI, USA), as described elsewhere [18]. Fibrinogen concentrations in cell lysates or culture media from the selected clones were measured by ELISA, and the assembly of fibrinogen and/or synthesis of three polypeptide chains in the cell lysate was analyzed by SDS-PAGE and a Western blot analysis, as described previously [23].

### 4.6. Statistical Analysis

The significance of differences in fibrinogen synthesis and secretion between mutant Bβ- and normal Bβ-CHO cell lines was analyzed using a one-way Mann–Whitney U test. A difference was considered to be significant when *p* < 0.05.

## Abbreviations

CHO	Chinese hamster ovary
RT-PCR	Reverse transcriptase-polymerase chain reaction
SDS-PAGE	Sodium dodecyl sulfate-polyacrylamide gel electrophoresis
ELISA	Enzyme-linked immunosorbent assay

## References

[1] Weisel J.W. (2005). Fibrinogen and fibrin. Adv. Protein. Chem..

[2] Weisel J.W., Litvinov R.I. (2013). Mechanisms of fibrin polymerization and clinical implications. Blood.

[3] Kant J.A., Fornace A.J., Saxe D., Simon M.I., McBride O.W., Crabtree G.R. (1985). Evolution and organization of the fibrinogen locus on chromosome 4: Gene duplication accompanied by transposition and inversion. Proc. Natl. Acad. Sci. USA.

[4] Acharya S.S., Dimichele D.M. (2008). Rare inherited disorders of fibrinogen. Haemophilia.

[5] Korte W., Poon M.C., Iorio A., Makris M. (2017). Thrombosis in Inherited Fibrinogen Disorders. Transfus. Med. Hemother..

[6] Bornikova L., Peyvandi F., Allen G., Bernstein J., Manco-Johnson M.J. (2011). Fibrinogen replacement therapy for congenital fibrinogen deficiency. J. Thromb. Haemost..

[7] Groupe d’Etude sur 1’Hémostase et la Thrombose, Base de Données des Variants du Fibrinogène GFHT Web Site. http://site.geht.org/site/Pratiques-Professionnelles/Base-de-donnees-Fibrinogene/Database-English-Version/Fibrinogen-variants-Database-_79_.html.

[8] Asselta R., Duga S., Spena S., Peyvandi F., Castaman G., Malcovati M., Mannucci P.M., Tenchini M.L. (2004). Missense or splicing mutation? The case of a fibrinogen Bbeta-chain mutation causing severe hypofibrinogenemia. Blood.

[9] Tompson S.W., Young T.L. (2017). Assaying the Effects of Splice Site Variants by Exon Trapping in a Mammalian Cell Line. Bio-Protocol.

[10] Casini A., Lukowski S., Quintard V.L., Crutu A., Zak M., Regazzoni S., de Moerloose P., Neerman-Arbez M. (2014). FGB mutations leading to congenital quantitative fibrinogen deficiencies: An update and report of four novel mutations. Thromb. Res..

[11] Brennan S.O., Davis R.L., Lowen R., Ruskova A. (2009). Deletion of five residues from the coiled coil of fibrinogen (Bbeta Asn167_Glu171del) associated with bleeding and hypodysfibrinogenemia. Haematologica.

[12] Hanss M., Ffrench P., Vinciguerra C., Bertrand M.A., Mazancourt P. (2005). Four cases of hypofibrinogenemia associated with four novel mutations. J. Thromb. Haemost..

[13] Brennan S.O., Laurie A.D., Bell J.A. (2016). Novel FGB mutation Bβ240Cys→Arg confirms importance of the Bβ211-240 disulphide for plasma expression of fibrinogen. Thromb. Res..

[14] Zhang J.Z., Redman C.M. (1996). Assembly and secretion of fibrinogen. Involvement of amino-terminal domains in dimer formation. J. Biol. Chem..

[15] Hanss M., Pouymayou C., Blouch M.T., Lellouche F., Ffrench P., Rousson R., Abgrall J.F., Morange P.E., Quélin F., de Mazancourt P. (2011). The natural occurrence of human fibrinogen variants disrupting inter-chain disulfide bonds (A{alpha}Cys36Gly, A{alpha}Cys36Arg and A{alpha}Cys45Tyr) confirms the role of N-terminal A{alpha} disulfide bonds in protein assembly and secretion. Haematologica.

[16] Platè M., Asselta R., Spena S., Spreafico M., Fagoonee S., Peyvandi F., Tenchini M.L., Duga S. (2008). Congenital hypofibrinogenemia: Characterization of two missense mutations affecting fibrinogen assembly and secretion. Blood Cells Mol. Dis..

[17] Terasawa F., Fujita K., Okumura N. (2005). Residue gamma153Cys is essential for the formation of the complexes Aalphagamma and Bbetagamma, assembly intermediates for the AalphaBbetagamma complex and intact fibrinogen. Clin. Chim. Acta.

[18] Soya K., Takezawa Y., Okumura N., Terasawa F. (2013). Nonsense-mediated mRNA decay was demonstrated in two hypofibrinogenemias caused by heterozygous nonsense mutations of FGG, Shizuoka III and Kanazawa II. Thromb. Res..

[19] Terasawa F., Kamijyo Y., Fujihara N., Yamauchi K., Kumagai T., Honda T., Shigematsu S., Okumura N. (2010). In vitro transcription of compound heterozygous hypofibrinogenemia Matsumoto IX; first identification of FGB IVS6 deletion of 4 nucleotides and FGG IVS3-2A>G causing abnormal RNA splicing. Clin. Chim. Acta.

[20] Mukai S., Nagata K., Ikeda M., Arai S., Sugano M., Honda T., Okumura N. (2016). Genetic analyses of novel compound heterozygous hypodysfibrinogenemia, Tsukuba I: FGG c.1129+62_65 del AATA and FGG c.1299+4 del A. Thromb. Res..

[21] Ikeda M., Arai S., Mukai S., Takezawa Y., Terasawa F., Okumura N. (2015). Novel heterozygous dysfibrinogenemia, Sumida (AαC472S), showed markedly impaired lateral aggregation of protofibrils and mildly lower functional fibrinogen levels. Thromb. Res..

[22] Mukai S., Ikeda M., Takezawa Y., Sugano M., Honda T., Okumura N. (2015). Differences in the function and secretion of congenital aberrant fibrinogenemia between heterozygous γD320G (Okayama II) and γΔN319-ΔD320 (Otsu I). Thromb. Res..

[23] Okumura N., Terasawa F., Tanaka H., Hirota M., Ota H., Kitano K., Kiyosawa K., Lord S.T. (2002). Analysis of fibrinogen gamma-chain truncations shows the C-terminus, particularly gammaIle387, is essential for assembly and secretion of this multichain protein. Blood.

